# Relationship of Anterior Knee Pain with Quadriceps Angle and Anthropometric Measurements in an Asian Female Population

**DOI:** 10.5704/MOJ.2207.012

**Published:** 2022-07

**Authors:** KY Phatama, SPP Isma, LK Devi, LD Siahaan, A Pribadi, AS Pradana, E Mustasir, M Hidayat

**Affiliations:** Department of Orthopaedic and Traumatology, Universitas Brawijaya, Malang, Indonesia

**Keywords:** quadriceps angle, Q-angle, anterior knee pain, anthropometry

## Abstract

**Introduction:**

Quadriceps angle (Q-angle) is the angle formed from the intersection of quadriceps femoris muscle and patellar tendon. Q-angle can be used to assess mechanical aspects of the patellofemoral discomfort and attracts considerable attention from clinicians. Anterior knee pain (AKP) is defined as mild pain localised around the peripatellar area exacerbated by physical activity such as climbing stairs, squatting, jumping, running, and/or sitting with the knee flexed for long periods. A higher Q-angle is one of the risk factors for AKP. In addition, several studies have found a relationship between anthropometric parameters, especially body mass index (BMI), to the occurrence of AKP. This study aims to determine the Q-angle and several anthropometric parameters between the population with AKP and without AKP in the adult population in Indonesia.

**Materials and methods:**

This cross-sectional, analytic experimental study was performed on female population divided into the comparison group consisted of 40 patients aged between 18-25 years with right side anterior knee pain (Kujala score <82) and the control group consisted of 40 patients aged between 18-25 years without anterior knee pain (Kujala score >82). Both groups had to be right-handed and had no history of knee fractures, knee surgery, congenital anomalies, or obesity. The Q-angle of the right knee was measured in all participants using a universal goniometer.

**Results:**

The mean Q-angle in the comparison group was 20.60±1.26°, and in the control group was 14.85±0.99°. All of these differences were statistically significant (p<0.001). There was no significant correlation between anthropometric parameters (body weight, body height, and body mass index) with AKP.

**Conclusion:**

The Q-angle can be used to predict the risk of AKP in the female population. The greater the Q-angle the higher the risk of AKP. Anthropometric measurements cannot predict the risk of AKP.

## Introduction

Anterior knee pain (AKP) or patella femoral pain syndrome (PFPS) is defined as mild pain localised around the peripatellar area aggravated by physical activity such as climbing stairs, squatting, jumping, running, and/or sitting with the knee flexed for an extended period. This disorder accounts for 25% of all knee pain found in sports medicine clinics. AKP is the most common diagnosis among young and physically active populations, affecting 25% of athletes, with >70% are aged between 16 and 25 years^1,2^. Some of the most common causes of AKP include patellofemoral malalignment, overuse, and trauma. One concept that can be used to assess patellar malalignment is the Q-angle^2^. Various studies showed a significant relationship between AKP and Q-angle. However, the available research currently limited and only comes from western populations with different body morphologies and physical activity from Southeast Asian populations, so the validity of this method is still unknown^1^.

**Fig 1:**
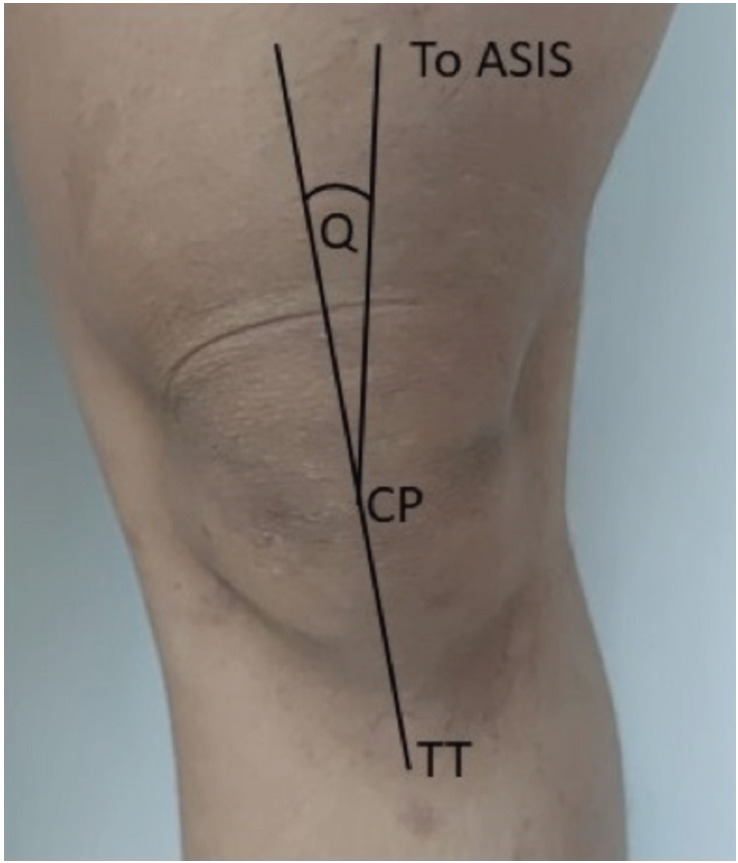
Q-angle measurement. ASIS – anterior superior iliac spine; CP – Centre of Patella; TT – Tibial Tuberosity; Q – Quadriceps angle.

The Q-angle is a vector index for the combined pull of the extensor and patellar tendon mechanisms. The Q-angle was initially introduced by Brattstrom (1964) and used as an indicator for dysfunction of the patellofemoral joint. To measure Q-angle, the physician assesses the angle formed from the intersection of two lines. The first line connects the anterior superior iliac spine (ASIS) to the centre patella (CP), and the second line connects the tibial tubercle (TT) to the CP (illustration shows in (Fig. 1). An increased Q-angle is considered an indication of a misalignment of the extensor mechanism triggered by knee overuse injury, which is associated with patellofemoral pain syndrome, knee joint hypermobility, patellar chondromalacia, recurrent patellar subluxation, and tearing of the anterior cruciate ligament (ACL)^3,4^.

AKP diagnosis is assessed based on the anterior knee pain scale (AKPS), better known as the Kujala Scale. Kujala scale contains 13 points self-report questionnaire on the knee to assess the patient’s clinical history with pain in the anterior knee. Kujala scale documents responses to six activities that are specifically related to AKP (walking, running, climbing stairs, jumping, squatting, and sitting with bent knees) as well as symptoms such as limping, inability to bear weight on a sore knee, swelling, abnormal knee movements, muscle atrophy, and limited bending of the knee. Kujala scale also records the duration of symptoms in the sore knee. The maximum value of this scale is 100, and a decrease in value indicates a decrease in knee function.

In addition, several studies have found a relationship between anthropometric parameters, especially body mass index (BMI), on the incidence of AKP, which an increased BMI has been shown to increase the risk of developing AKP. Unfortunately, these studies use a different research sample from the anthropometry of the Indonesia population so that the results do not necessarily match the conditions in Indonesia. After looking at the previously mentioned factors and the limitations of those studies, this study aims to assess the relationship between the incidence of AKP and the Q-angle and anthropometric parameters (BMI, body height, and body weight) in an Asian Female Population. We have decided to use females as our sample as females are more prone to AKP due to the greater Q-angle. The ability to predict AKP in females can provide physicians with preventive measures for AKP. In addition, previous studies use a sample in the western population, which differs from the Asian population. The female population samples in this study are Asians and we aim to add information on the relationship of anterior knee pain with quadriceps angle and anthropometric measurements, especially in the Asian population.

## Materials and Methods

The research design was an observational analytic study with a cross-sectional approach. The subjects were Indonesia female citizens who meet the inclusion and exclusion criteria. They must be right-handed and do not have a history of congenital anomalies or injuries, abnormalities, fractures and surgery on the knee and do not have a history of obesity. This research was conducted in the Faculty of Medicine, Universitas Brawijaya Fakultas Kedokteran, from January to February 2021 by taking primary data from samples that met the criteria. Eighty women were divided into two groups consisting of a case group and a control group. The case group involved 40 women aged 18-25 years with right side Anterior Knee Pain (AKP) who had a Kujala score <82. The control group consisted of 40 women aged 18-25 years without Anterior Knee Pain (AKP) with a Kujala score >82. The objective was to determine the relationship between Q-angle and anthropometric parameters (BMI, body height, and body weight) and the incidence of AKP. The dependent variables in this study were knee with AKP (Kujala score <82) and knee without AKP (Kujala score >82), while the independent variables were Q-angle and anthropometric parameters. A female student population was utilised as they are considered a homogenous sample population with a moderate-low activity level. This research was conducted under the permission of the Ethical Committee of Medical Research Faculty of Medicine, Universitas Brawijaya Fakultas Kedokteran.

Q-angle measurement procedure were taken after the participants who accepted to take part in this study signed an Informed Consent Form. The Q-angle measurement were evaluated by the same person every time by positioning the patient in a supine position. Subjects were previously asked to be barefoot and wear suitable clothing such as shorts or underwear so that the anatomical points to be seen and measured could be seen more clearly. Both of the patient’s limbs are in an extended position with the relaxation of the quadriceps muscles. The feet are stuck to each other in an anatomical position with the toes facing up.

The following step is to identify the anatomical points of the patient by placing several markers. The anatomical points were ASIS, CP, and the midpoint of TT. In order to find the centre patella precisely, measuring using a caliper was mandatory. CP is defined as the meeting point of the vertical and horizontal lines of the patella. The highest prominence point of the tibia tubercle is the central TT. The first line was drawn from the CP to the ASIS using a flexible ruler. Next, the second line was drawn from the TT to the CP upwards until it intersects the first line. The angle formed between the intersection of these two lines is called Q-angle and is measured by a goniometer.

Body height, body weight, BMI (weight (kg)/body height (m2), and waist-hip ratio were measured to determine the obesity classification. Pelvic width was measured by calculating the pelvic interspinous distance (cm) at the umbilicus level when the patient was in the mid expiratory position. Besides, hip circumference was measured by measuring the widest size of the greater trochanter. Finally, the waist-hip ratio was calculated using these two parameters. In obese patients (BMI >3) with the waist-hip ratio ≥0.85 for women was classified as central obesity, where the waist-hip ratio <0.85 was classified as lower body obesity.

Data analysis was done by manual data collection in Microsoft Excel®. Then, the collected data was processed through the normality test using the Kolmogorov-Smirnoff test. If the data were normally distributed (p>0.05), the comparison between Q-angle and anthropometric variables (BMI, BW, TB) of the case group and control group would be tested using an independent T-test. On the contrary, comparisons will be made using the Mann-Whitney U test if the data was not normally distributed. In addition, the correlation between Q-angle and anthropometric variables will be tested using Pearson product correlation if the data were normally distributed or using Spearman correlation if it was not normally distributed.

## Results

This study used 80 samples of Indonesia students consisting of 40 females with AKP and 40 females without AKP. The Table I shows the characteristics and the comparison of the research sample.

**Table I:** Comparison and correlation between Right leg Q-angle with anthropometry in the population with AKP and without AKP

Characteristics	Population with anterior knee pain (n=40)	Population without anterior knee pain (n=40)	P-value
Body weight	53.75±8.52	54.03±8.42	0.12
Body height	156.12±3.76	158.02±4.92	0.93
Body Mass Index	22.09±3.25	21.63±3.28	0.43
Right leg Q-angle	20.60±1.26	14.85±0.99	<0.001*
**Correlation**	**P-value (Spearman Correlation)**		
Body height – Right Q-Angle	0.28		
Body weight – Right Q-Angle	0.08		
Body mass index – Right Q-Angle	0.14		

From the population characteristics table, it can be seen that there is no significant difference in terms of characteristics (weight, height, and BMI) between the population with AKP and the population without AKP (p-value >0.05). This result may be caused by the homogeneity of the population in this study (Universitas Brawijaya students). However, the Kolmogorov-Smirnov test of these variables shows a p-value <0.05, which indicates that the distribution of data from all variables is not normal, so that the comparison between the two groups in this study used the Mann-Whitney test.

Mann-Whitney test was performed to determine the ratio of Q-angle between groups of women with right side AKP and women without AKP. The following table (Table I) compared Q-angle on the right leg in the population without AKP and the population with AKP.

**Fig 2:**
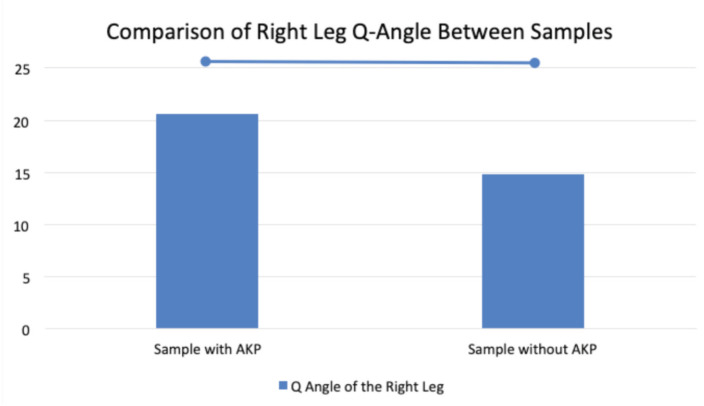
Comparison of Right leg Q-angle in the population with AKP and the population without AKP.

Based on Fig. 2, it was shown that the average right leg Q-angle of the sample with AKP is 20.60±1.26, while the right leg Q-angle of the sample without AKP is 14.85±0.99. Furthermore, the p-value from the Mann-Whitney test was <0.0001, which indicates a significant difference in Q-angle between samples with AKP and samples without AKP.

Spearman test was performed to determine the correlation between the right leg Q-angle and anthropometry. Table I shows the correlation test results between the right leg Q-angle and anthropometry (body weight, body height, and body mass index).

**Fig 3:**
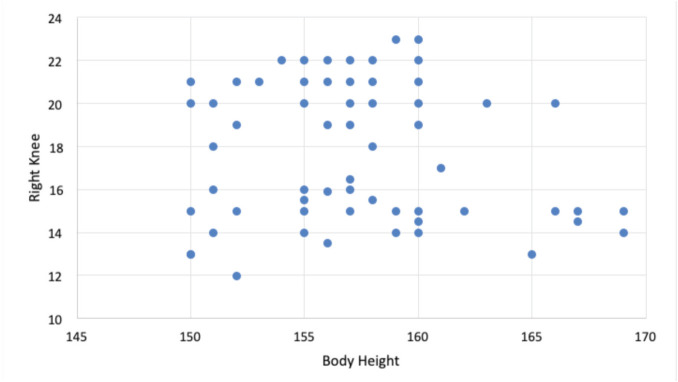
The distribution of right leg Q-angle in relation to body height.

The test results showed no significant correlation between the right leg Q-angle and anthropometry. The p-value between right leg Q-angle and height was 0.28, which is more than 0.05 (p>0.05), indicating no relationship of Q-angle and height between population with AKP and without AKP. Fig. 3 shows the distribution of the Q angle on the right leg with its relationship to body weight.

**Fig 4:**
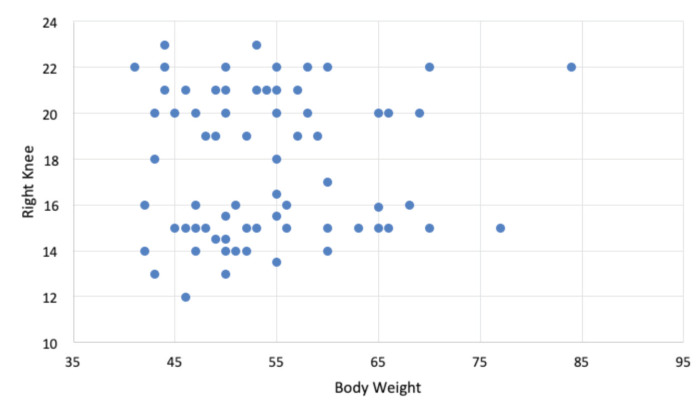
The distribution of right leg Q-angle in relation to body weight.

In addition, there was no significant correlation between Q-angle on the right leg and body weight (p=0.08). P-value more than 0.05 (p>0.05) indicates no significant Q-angle and body weight relationship between the sample with AKP and without AKP. The results were considered statistically significant if the p-value was <0.05. Fig. 4 shows the distribution of the Q angle on the right leg with its relationship to body height.

**Fig 5:**
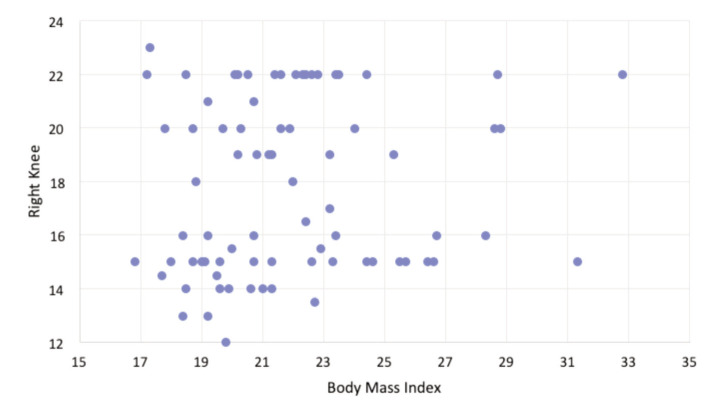
The distribution of right leg Q-angle in relation to body mass index.

There was also no significant correlation between Q-angle on the right leg and BMI (p=0.14). The P-value was more than 0.05 (p>0.05), indicating no significant relationship between Q-angle and BMI in the population with AKP and without AKP. Fig. 5 shows the distribution of the Q angle on the right leg with its relationship to BMI.

## Discussion

The quadriceps angle (Q-angle), formed between the quadriceps muscle and the patellar tendon, is a clinically significant parameter that affects the biomechanics of the quadriceps of the knee and affects the movement of the patella on the knee. Q-angle is an index parameter for diagnosing knee problems, one of which is anterior knee pain.

The result shows that the average right leg Q-angle of the sample with AKP is higher than the right leg Q-angle of the sample without AKP with a p-value <0.0001, which indicates a significant difference in Q-angle between samples with AKP and samples without AKP.

Based on other previous studies, a high Q-angle value will cause an abnormally high valgus angle. This abnormally high valgus angle will affect the biomechanics of the patellofemoral joint through increased lateral traction of the quadriceps muscle on the patella. This problem was causing mal-tracking and overpressure on the patellofemoral joint and ultimately resulting in anterior knee pain.

This result was also consistent with a preliminary study in Faculty of Medicine, Universitas Brawijaya, conducted on 800 knees showing that the mean Q-angle in men ranged from 12.81±1.81 on the right leg, and 13.06±1.78 on the left leg. Meanwhile, the average Q-angle for women was 14.95±1.28 on the right leg, and 14.98±1.28 on the left leg. A larger Q-angle in women can increase the incidence of AKP in women. A similar result was obtained in the study conducted by Kumar *et al*, which stated that there was a significant difference in the Q-angle value between the anterior knee pain sample (17.9±4.2) and the normal sample (14.3±3.6)5. In addition, another study by Caylor *et al* also showed the same results. The Q-angle on the AKP sample was 12.4±5.1, while the normal population was 11.1±5.5 with 1.3 Q-angle differences6. Finally, another study initiated by Moss *et al* also reported a higher Q-angle value in the AKP population (17.1°) than in the normal population (15.2°)7.

There are several references to the normal value of Q-angle that researchers have documented, some consider 10° to be normal and 15°-20° to be abnormal, other opinions say that value lower than 10° is considered problematic8. Women have a bigger Q-angle than men because women have a wider pelvis (gynecoid), a shorter femur, or a more lateral tibia tuberosity9,10. A study stated that women have a 2.23 times greater risk than men to develop AKP. Several other factors may play a role in this increased risk, including a larger knee valgus angle, a greater hip adduction moment, a smaller knee valgus moment, and a smaller knee flexion angle. Female populations are also found to have weaker quadriceps, hip external rotators, hip extensors, and hip abductors muscle strength than men11.

From the test results we can see that there is no significant correlation between the right leg Q-angle and anthropometry. From this study anthropometry such as height, weight, and BMI did not have relationship with Q-angle between population with AKP and without AKP. In contrast with several previous studies that found a significant negative correlation between Q-angle and height, the taller a person is, the smaller the Q-angle value for that person, and vice versa12-15. But for correlation between Q-angle on the right leg and weight, the results of this study are in parallel with the studies by Choudhary *et al* and Khasawneh *et al*, who found no significant correlation between Q-angle and body weight12,15.

There was also no significant correlation between Q-angle on the right leg and BMI in the population with AKP and without AKP. These results were consistent with the study conducted by Kumar *et al*, who found no significant correlation between Q-angle and BMI. However, there was another study by Prakash *et al*, who found a significant correlation between Q-angle and BMI. This result may be due to a large number of samples with obesity in the study. Meanwhile, our sample’s BMI did not reach obesity criteria with the mean BMI in the population with AKP of 22.09±3.25 and without AKP of 21.63±3.285,16. Similar result was showed in a systematic review by Hart *et al* that found higher BMI increases the risk of developing AKP. In contrast with BMI, weight and height did not have a significant effect of AKP17. Arrebola *et al* also agreed that BMI is a risk factor for AKP18.

Although it provided satisfactory results, this study had several limitations. First, this study did not evaluate the relationship between gender and the Q-angle. Second, we also did not evaluate left and right-side Q-angle differences. Third, we did not evaluate the difference in Q-angle between ethnic groups in Faculty of Medicine, Universitas Brawijaya. Previous studies have found that ethnicity affects the Q-angle value due to differences in physical appearance in each ethnicity, such as hip shape and height12,19. Therefore, further research analysing this relationship is still needed.

## Conclusion

The Q-angle can be used to predict the risk of AKP in the female population. The greater the Q-angle the higher the risk of AKP. Meanwhile, anthropometric measurements cannot be used to predict the risk of AKP in the female population.
